# The antiretroviral agent saquinavir enhances hTERT expression and telomerase activity in human T leukaemia cells in vitro

**DOI:** 10.1186/1756-9966-32-38

**Published:** 2013-06-08

**Authors:** Riccardo Adamo, Alessandro Comandini, Angelo Aquino, Laura Bonmassar, Loredana Guglielmi, Enzo Bonmassar, Ornella Franzese

**Affiliations:** 1 Department of Systems Medicine, Pharmacology Section, University of Rome Tor Vergata, Rome, Italy; 2 Laboratory of Molecular Oncology, Istituto Dermopatico dell’Immacolata-IRCSS, Via dei Monti di Creta 104, 00167, Rome, Italy; 3 CNR-Cellular Biology and Neurobiology Institute, Via del Fosso di Fiorano 64, 00143, Rome, Italy; 4 Institute of Translational Pharmacology (IFT) National Council of Research Rome, Via Fosso del Cavaliere 100, Rome, Italy

**Keywords:** Leukaemia, Telomerase, hTERT, Saquinavir, c-Myc

## Abstract

**Background:**

Saquinavir, a protease inhibitor utilized in HIV infection, shows antitumor activity in various experimental models. In previous studies performed in our laboratory the drug was found to induce a substantial increase of telomerase activity in normal peripheral blood mononuclear cells. Aim of the present investigation was to test whether saquinavir was able to increase telomerase activity and the expression of the catalytic subunit of telomerase, hTERT, in human malignant hematopoietic cells.

**Methods:**

Human Jurkat CD4^+^ T cell leukaemia cell line was used throughout the present study. The antiproliferative effect of saquinavir was tested by the MTT assay. Telomerase activity was determined according to the telomeric repeat amplification protocol. The expression of hTERT mRNA was semi-quantitative evaluated by RT-PCR amplification and quantitative Real Time PCR. The binding of the transcription factor c-Myc to its specific E-Box DNA binding-site of *hTERT* promoter was analyzed by Electophoretic Mobility Shift Assay (EMSA). The amount of c-Myc in cytoplasm and nucleus of leukemia cells was determined by Western Blot analysis, and c-Myc down-regulation was obtained by siRNA transfection.

**Results:**

Saquinavir produced a substantial increase of telomerase activity in Jurkat cells in vitro without increasing but rather reducing target cell proliferation rate. Telomerase up-regulation appeared to be the result of enhanced expression of hTERT. Saquinavir-mediated up-regulation of hTERT gene was the result of the increased binding of proteins to the E-Box sequence of the promoter. Moreover, saquinavir amplified the expression of c-Myc especially in the nuclear cell fraction. The direct influence of saquinavir on this transcription factor was also demonstrated by the antagonistic effect of the drug on siRNA induced c-Myc suppression. Since c-Myc is the main responsible for hTERT transcription, these findings suggest that the main mechanism underlying saquinavir-induced telomerase activation is mediated by c-Myc up-regulation.

**Conclusions:**

Saquinavir augments hTERT expression while inhibiting leukemic cell growth. Experimental evidences show that this effect is mediated by saquinavir-influenced increase of c-Myc levels. This could have relevance in terms of enhanced hTERT-dependent tumor cell immunogenicity and suggests new paharmacological approaches interfering with c-Myc dependent pathways.

## Background

Several protease inhibitors (PI) have been long term FDA-approved agents for the treatment of human immunodeficiency virus (HIV-1) infection [1]. More recently, these compounds [2-4] including the NO derivative of saquinavir [5,6], have shown noticeable antitumor activity, that is distinct from their antiviral properties. This finding has been originated by the observation that patients taking antiretroviral protease inhibitors showed a lower incidence of infection-associated malignancies leading to the hypothesis that these drugs could have antineoplastic properties [7]. Initially this effect was attributed mostly to the PI-induced immune reconstitution. Actually, we demonstrated that saquinavir was able to contrast T cell senescence by inducing up regulation of telomerase and an increased capability to produce IFN-γ following stimulation [8,9].

In nude mice, PIs, such as saquinavir and indinavir were shown not only to be able to block the development but also to induce the regression of angioproliferative sarcoma-like lesions [10]. These neoplasms were originated by primary human Kaposi sarcoma cells stimulated by basic fibroblast growth factor (bFGF) and vascular endothelial growth factor (VEGF). Thereafter, antitumor activity of saquinavir or indinavir was also demonstrated in “viral free” tumor models, consisting in nude mice bearing human neoplasias, including highly aggressive lung, breast, colon and hepatic carcinomas [4]. PIs are capable of targeting both matrix metalloproteinases [4] and the proteasome [11]. Moreover, Timeus et al. demonstrated that saquinavir suppresses imatinib-sensitive and imatinib-resistant chronic myeloid leukaemia cells [12]. In this case, saquinavir, showed dose- and time-related anti-proliferative and pro-apoptotic effects, particularly on the imatinib-resistant lines. Furthermore, in this experimental model the activity of saquinavir was significantly amplified by combination with imatinib itself. The direct antitumor effects of saquinavir was confirmed by McLean et al. [7] who demonstrated how the drug is able to induce endoplasmic reticulum stress, autophagy, and apoptosis in human ovarian cancer cells in vitro.

Telomerase is a specialized RNA template/reverse transcriptase enzymatic complex which synthesizes and adds TTAGGG repetitive nucleotide sequences to the end of chromosomes compensating for telomeric loss occurring at each cell replication [13]. Most differentiated somatic cells deactivate telomerase and undergo telomere shortening. However, the enzyme is reactivated in stimulated lymphocytes and proliferating stem cells, and is constitutively expressed and functioning in malignant cells that acquire the “immortal” phenotype. For this reason, human telomerase reverse transcriptase (hTERT) is considered a universal, although not specific, tumor-associated antigen [14-16]. Actually, hTERT-derived peptides are presented by major histocompatibility complex (MHC) class I alleles to T lymphocytes and activate a specific immune response with a potential role in cancer immune therapy. Indeed, CD8^+^ cytotoxic T lymphocytes (CTLs) specific for the hTERT-derived antigenic epitopes lyse hTERT-positive tumors of different origin [16]. These findings identify hTERT as an important tumor antigen applicable for anti-cancer vaccine strategies [17].

Previous studies conducted in our laboratory, demonstrated that saquinavir was able to increase telomerase activity in T lymphocytes [8,9], suggesting a role for this PI against T cell senescence, through telomerase activation.

In the present study we investigated the “in vitro” effect of saquinavir on telomerase activity of Jurkat CD4^+^ T leukaemia cells. The results confirmed an anti-proliferative effect of saquinavir also in this model and pointed out that the drug was able to up-regulate telomerase activity and hTERT expression at transcriptional level, most likely through c-Myc accumulation. Saquinavir-mediated inhibition of cell growth and increase of telomerase activity show two different aspects of its prospective role in malignant cell control. In fact, from one side saquinavir possesses direct tumor suppressive activity and from the other side, it could be potentially able to increase hTERT-dependent tumor cell immunogenicity [16,17].

## Materials and methods

### Cell line and culture condition

Human Jurkat CD4^+^ T cell leukaemia cell line was obtained from the American Type Culture Collection (ATCC, Rockville, MD, USA) and was passaged for less than six months continuously and routinely checked for mycoplasma contamination. Cell line was cultured at 37°C in a 5% CO_2_ humidified atmosphere in RPMI-1640 (Gibco, Paisley, Scotland, UK), supplemented with 10% heat inactivated (56°C, 30 min) fetal calf serum (Gibco), 2 mM L-glutamine, and antibiotics (Flow Laboratories, McLean, VA, USA), hereafter referred to as “Complete Medium” (CM). Saquinavir was a kind gift from prof. C.F. Perno (University of Tor Vergata).

### MTT assay

50 × 10^3^ Jurkat cells suspended in 100 μl CM in 96-well tissue culture plates were treated with saquinavir or the drug vehicle DMSO as control and incubated at 37°C and 5% CO_2_. After 96 h of culture, 0.1 mg of MTT (in 20 μl of PBS) was added to each well and cells were incubated at 37°C for 4 h. Cells were then lysed with a buffer (0.1 ml/well) containing 20% SDS and 50% N,N-dimethylformamide, pH 4.7. After an overnight incubation, the absorbance was read at 570 nm using a 3550-UV microplate reader (Bio-Rad). Inhibition of proliferation of tumor cells by saquinavir obtained in 3 separated experiments has been expressed in terms of inhibitory concentration 50% (IC50) along with confidence interval calculated as previously described [18].

### TRAP assay

Telomerase activity was determined according to the telomeric repeat amplification protocol [19]. Briefly, telomerase activity was assayed in whole cell extracts. Cell samples for detection of telomerase activity were collected at the time intervals indicated in the results. Cells were washed in PBS and lysed in ice-cold extraction buffer containing 0.5% 3[(cholamidopropyl)-dimethyl-ammonium]-1-propanesulfonate, 10 mM Tris–HCl (pH 7.5), 1 mM MgCl2, 1 mM EGTA, 5 mM β-mercaptoethanol, 0.1 mM [4(2-aminoethyl)-benzenesulfonyl fluoride] hydrochloride, and 10% Glycerol (Sigma). Extracts from 500 Jurkat cells were used for TRAP assay. TRAP assay was performed in 50 μl of reaction mixture [20 mM Tris–HCl (pH 8.3), 68 mM KCl, 1.5 mM MgCl2, 1 mM EGTA, 0.05% Tween 20, 0.1 mg of TS (5’-AATCCGTCGAGCAGAGTT) primer, 0.5 mM T4 gene 32 protein, 10 mM deoxynucleotide triphosphate, 2 units of Taq polymerase (Promega,Madison, WI, USA), and 2 μCi of (γ-32P)dCTP (3000 CI/mmol; DuPont NEN Research Products, Boston, MA)]. Each reaction was carried out in a single PCR tube containing 100 ng of CX oligonucleotide 5’ -(CCCTTTA)3CCCTAA (Biogen, Rome, Italy), sealed at the bottom of the tube by a wax barrier. Samples were incubated at 22°C for 20 minutes to allow telomerase to extend TS primer, followed by a 31-cycle PCR amplification (Perkin Elmer Corp., Norwalk, CT) of the telomeric products. Forty μl of the PCR products were run on 10% non-denaturing acrylamide gels. Gels were fixed in 0.5 M NaCl, 50% Ethanol, and 40 mM Sodium Acetate (pH 4.2) and then exposed to X-Ray Film (Kodak, Rochester, NY) at – 80°C.

The signal intensity of each band was measured and expressed in optical density (OD). The semi-quantitative analysis of telomerase activity was performed by adding the signals of the ladder products in each lane, corrected for the background.

### RT-PCR

The expression of hTERT mRNA was semi-quantitatively evaluated by RT-PCR amplification as described [20]. Briefly, hTERT mRNA was amplified using the primer pairs: 5’-CGGAAGAGTGTCTGGAGCAA-3’ and 5’- GGATGAAGCGGAGTCTGGA-3’ . Total RNA was isolated from the cells using Trizol (Invitrogen) according to the manufacturer’s protocol, and cDNA was synthesized from 1 μg of RNA using the cDNA Cycle kit (Invitrogen) with random primers. Typically, 2 μl aliquots of the reverse-transcribed cDNA were amplified by 28 cycles of PCR in 50 μl of buffer [10 mM Tris–HCl (pH 8.3), 2.5 mM MgCl_2_, and 50 mM KCl] containing 1 mM each of dATP, dGTP, dTTP, and ^32^P- dCTP (Amersham Biosciences, Amersham, United Kingdom), 2.5 units of Taq DNA polymerase (Promega, Madison, Wisconsin), and 0.2 mM primers. Each cycle consisted of denaturation at 94°C for 30 s, annealing at 60°C for 30 s and extension at 72°C for 45 s. The PCR products were resolved by electrophoresis in 7% polyacrylamide gels. The efficiency of cDNA synthesis from each sample was estimated by PCR using GAPDH specific primers: 5’ - GAAGGTGAAGGTCGGAGTC-3’and 5’-GAAGATGGTGATGGGATTTC-3’.

### Real time PCR

Briefly 2 × 10^6^ viable Jurkat T cells treated or not with saquinavir were harvested after 24 h incubation. Samples were resuspended in 1 ml Trizol (Ambion) and RNA samples were extracted according to the manufacturer's instructions. Two μg of RNA were purified by clearance of DNA traces using Turbo DNA-free kit (Applied Biosystems, Life Technologies, Monza, Italy). cDNA was synthesized using 2 μg of DNA-free RNA and TaqMan RT kit (Applied Biosystems), according to the manufacturer’s instructions. hTERT mRNA was quantitatively detected by real-time reverse transcription polymerase chain reaction (RT-PCR). For quantitative real time RT-PCR 5 μl (i.e. 2 μg) of cDNA/sample was amplified according to the manufacturer’s instructions (Applied Biosystems) on a Real-Time Stratagene MX3005P, using a TaqMan gene expression assay kit (Applied Biosystems, code # Hs00162669-m1). Levels of hTERT were normalized against GAPDH housekeeping expression (Applied Biosystems code # 4326317E). All real-time RT-PCR reactions were performed in triplicate. Normalized TERT expression (TERT/GAPDH) was calculated using the ΔΔCt method according to the supplier’s protocol.

### Electophoretic mobility shift assay (EMSA)

The binding of the transcription factor c-Myc to its specific downstream E-Box DNA binding-site from *hTERT* promoter was analyzed by EMSA [21]. In particular we analyzed the DNA oligonucleotide 5’- TCCTGCTGCGCACGTGGGAAGCCCT-3’, containing the downstream “CACGTG” E-Box sequence localized at position −34 of hTERT promoter. Nuclear extracts were obtained as previously described [22] from extracts of 2 × 10^2^ viable cells. Five micrograms of nuclear proteins/reaction were incubated with 30 000 cpm of ^32^P-γ-ATP (Amersham) end-labeled E-Box oligonucleotide extrapolated from hTERT promoter. Binding reactions were performed in a 10-μl volume for 20 min at room temperature in a buffer consisting of 5 mg/ml poly(dI– dC), 10mM Tris–HCl, 50mM NaCl, 0.5mM DDT, 0.5 mM EDTA, 1 mM MgCl2, 4% glycerol, pH 7.5 (Promega). For competition assays, 100-fold molar excess of c-Myc standard oligonucleotide (Promega) was used in the binding reaction (data not shown).

Protein–DNA complexes were resolved by 5% polyacrylamide gel electrophoresis (PAGE) at 4°C. Dried gels were exposed to X-Ray film (Amersham) at −70°C for 12 h.

### Western blot

For Western Blot analysis of whole cell extracts, cells were isolated at times indicated and lysates obtained by sonicating cells in 50 mM Tris–HCl pH 7.5, 2 mM EGTA, 0.1% triton X-100 buffer. Cytosol and nuclear extracts were prepared as previously described [22]. Lysates from 2 × 10^6^ cells were separated by gel electrophoresis on 10% sodium dodecyl sulphate-polyacrylamide gels and transferred to Hybond-P membranes (Amersham Pharmacia Biotech, Piscataway, NJ). Membranes were then probed with anti hTERT (Santa Cruz Biotech Inc.) and anti c-Myc (Cell Signalling) antibodies following the instructions provided by the manufacturers. All filters were probed with anti GAPDH (Santa Cruz) as loading control. Quality of nuclear extracts was analyzed using anti Histone H1 Ab (Upstate, Lake Placid, NY, USA). Analysis was performed using the ECL Plus Western detection kit (Amersham Pharmacia Biotech).

### c-Myc siRNA

To inhibit Myc expression we used a siRNA technology. The siRNA used were purchased from Qiagen: Hs_LOC731404_4 (#SI03528896) targeting c-Myc mRNA and AllStars (#1027280), a nonsilencing siRNA with no homology to any known mammalian gene, as negative control. For the transfection procedure, exponentially growing Jurkat cells were seeded in 24-well plates at a concentration of 2×10^5^ cells/well in 100 μl CM. Immediately cells were transfected with siRNA using the HiPerFect Transfection Reagent (Qiagen), according to a manufacturer’s specific protocol for Jurkat cells. Briefly, siRNAs were incubated in serum-free medium with HiPerFect Transfection Reagent for 10 min at room temperature. Subsequently, the mixture was added to each well and incubated for 6 h. Then, 400 μl of complete medium were added to each well and after 24 h the cells were treated with the drug for further 24 h. The final concentration of each siRNAs in each well was 75 nM.

### Data analysis and statistics

Band intensity of the experiments was quantified by bi-dimensional densitometry (Bio-Rad, Richmond, CA). Statistical significance was evaluated using student *t*-test analysis. This was performed taking into account the mean and standard deviation of optical densitometric values obtained in independent experiments. Differences were considered significant when *p values* were less than 0.05.

## Results

### Effect of saquinavir on “in vitro” Jurkat cell growth

**Figure 1:**
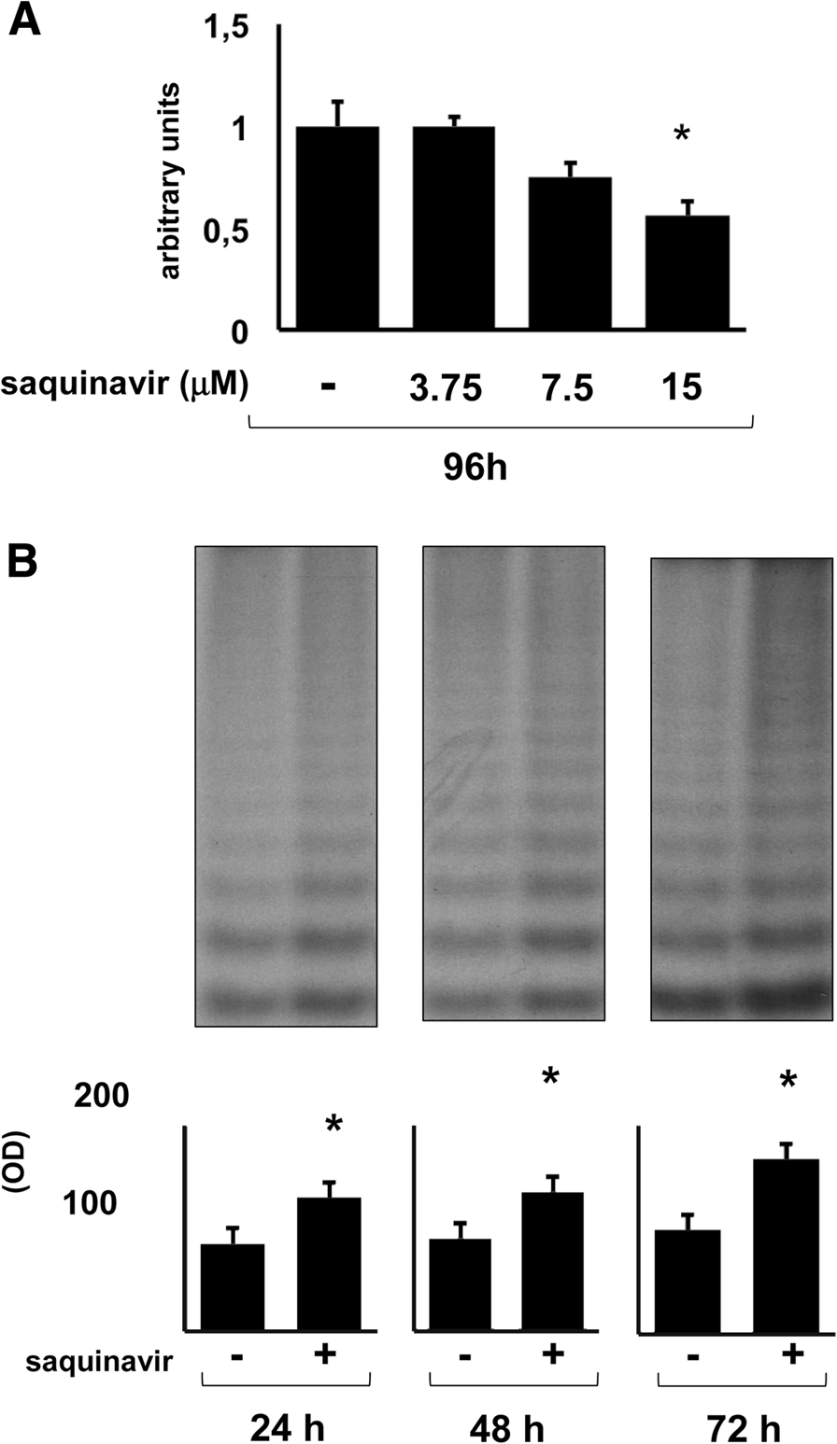
**Effect of saquinavir on cell growth and telomerase activity. A.** After 96 h, of culture MTT assay was performed as described in “Materials and Methods”, on Jurkat cells treated with saquinavir 3.75, 7.5 and 15 μM or DMSO as control. Saquinavir concentration which inhibited significantly cell viability (15 μM, p < 0,005), was close to the IC50 (i.e. 17. 36 μM, see “Results” section). The data are represented as percentage cell viability of the untreated cells. Each bar represents the mean ± SD of determinations from 3 independent experiments. Asterisk indicates p < 0.05. **B.** Representative blot of telomerase activity (TRAP Assay) of whole cell extracts from 500 viable Jurkat cells determined 24, 48 and 72 h following treatment with saquinavir. Graph shows the mean ± SD of OD obtained from pooled results of the effect of saquinavir (15 μM) on telomerase activity of Jurkat cell line from 3 separate experiments. All p values were calculated using Student’s t-test. Asterisk indicates p < 0.05.

Saquinavir has shown dose- and time-related anti-proliferative and pro-apoptotic effects on different tumors [3,4]. Graded concentrations of saquinavir (from 3.75 to 15 μM) were added to Jurkat cell suspension as described in Material and Methods. The effect of saquinavir on Jurkat cell growth has been evaluated using the MTT assay, performed after 96 h of incubation with the antiretroviral agent. The results obtained from 3 pooled independent experiments and shown in Figure 1A, indicate that the IC 50 was 17.36 μM, with a confidence interval corresponding to 8.93 and 25.79 μM.

### Influence of saquinavir on telomerase activity of Jurkat cell line

Telomerase is a specialized RNA template-containing reverse transcriptase able to compensate for telomeric loss occurring at each cell replication, which is reactivated in tumor cells [13]. In previous studies we found that saquinavir was able to increase telomerase in T cells [8,9]. Here we analyzed the effect of saquinavir on telomerase activity of Jurkat cells after 24, 48 and 72 h of treatment. Based on the results obtained in terms of cell growth inhibition, we decided to use the concentration of 15 μM of the agent throughout the next steps of our study. We found that the protease inhibitor was able to induce up-regulation of telomerase activity, from 24 h to 72 h of cell exposure (Figure 1). Similar results were obtained by pooling data obtained from 3 independent experiments in correspondence of all analyzed time intervals (Figure 1B).

### Influence of saquinavir on telomerase catalytic subunit hTERT expression

**Figure 2:**
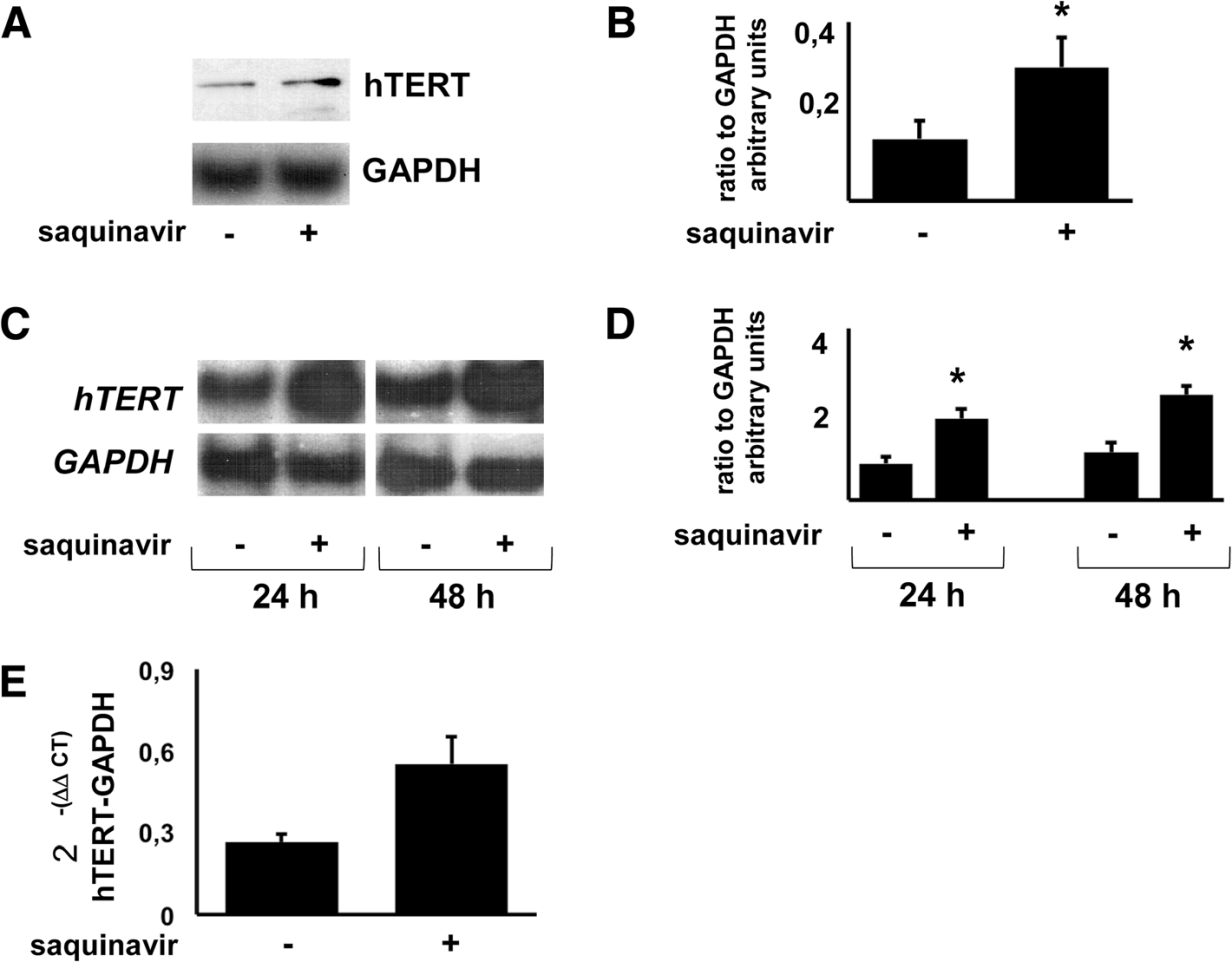
**Effect of saquinavir on hTERT expression. A.** Representative experiment showing the effect of saquinavir (15 μM) on hTERT expression tested on whole cell extracts from 2×10^6^ viable CD4^+^ Jurkat cells 48 h following treatment (Western Blot). Gel loading control was based on GAPDH expression. Saquinavir increases hTERT levels in Jurkat cells. **B**. Graph shows the mean ± SD of the ratio hTERT/GAPDH band intensity obtained by pooling the results from 3 independent experiments. **C**. Representative gel showing the effect of saquinavir on hTERT mRNA in Jurkat cell line, determined after 24 and 48 h of treatment, using RT-PCR. GAPDH was used as internal control. Saquinavir up-regulates hTERT mRNA transcription. **D.** Graphs show the mean ± SD of OD for 3 independent RT-PCR experiments. **E.** Effect of saquinavir on hTERT mRNA expression of Jurkat cells 24 hours following treatment analysed by quantitative real-time RT-PCR. Levels of hTERT are normalized against GAPDH housekeeping expression. The graph shows the difference in terms of gene expression working out the Delta Delta CT algorithm between TERT and the housekeeping GAPDH. Data shown are representative of 2 independent experiments. All p values were calculated using one-way paired Student’s t-test. Asterisk indicates p < 0.05.

A major mechanism regulating telomerase activity in human cells is transcriptional control of the telomerase catalytic subunit gene, hTERT [23]. Several transcription factors, including oncogene products (e.g. c-Myc) and tumor suppressor gene products (e.g. WT1 and p53), are able to control hTERT transcription [23]. Experiments were performed in order to estabilish whether the observed up-regulation of telomerase activity mediated by saquinavir was the consequence of an increased expression of the catalytic subunit hTERT. Therefore, cells were exposed to saquinavir for 48 h, lysed as described in Material and Method section and separated by SDS-PAGE. This time point was chosen after time course experiments were run in order to determine the best interval for this observation. Results exposed in Figure 2A show that saquinavir was able to increase hTERT total level in Jurkat cells. Therefore, it is reasonable to consider that the up-regulated levels of telomerase activity observed in drug-treated Jurkat cells could be the consequence of the increased levels of catalytic subunit hTERT. These results were confirmed by pooled data obtained from 3 different experiments (Figure 2B). This observation was also confirmed at transcriptional level. mRNA expression of hTERT was analyzed by semi-quantitative RT-PCR in Jurkat controls and in saquinavir-treated cells. Twenty-four and 48 hours after stimulation, RNA was extracted and RT-PCR assay was performed to detect hTERT mRNA. Saquinavir was able to up-regulate hTERT mRNA expression according to the results obtained in the experiment illustrated in Figure 2C and in the pooled results relative to 3 separate experiments (Figure 2D). These results were further confirmed by quantitative Real Time-PCR experiments performed after 24 hours following exposure to the drug and illustrated in Figure 2E.

### Influence of saquinavir on hTERT transcriptional control

**Figure 3:**
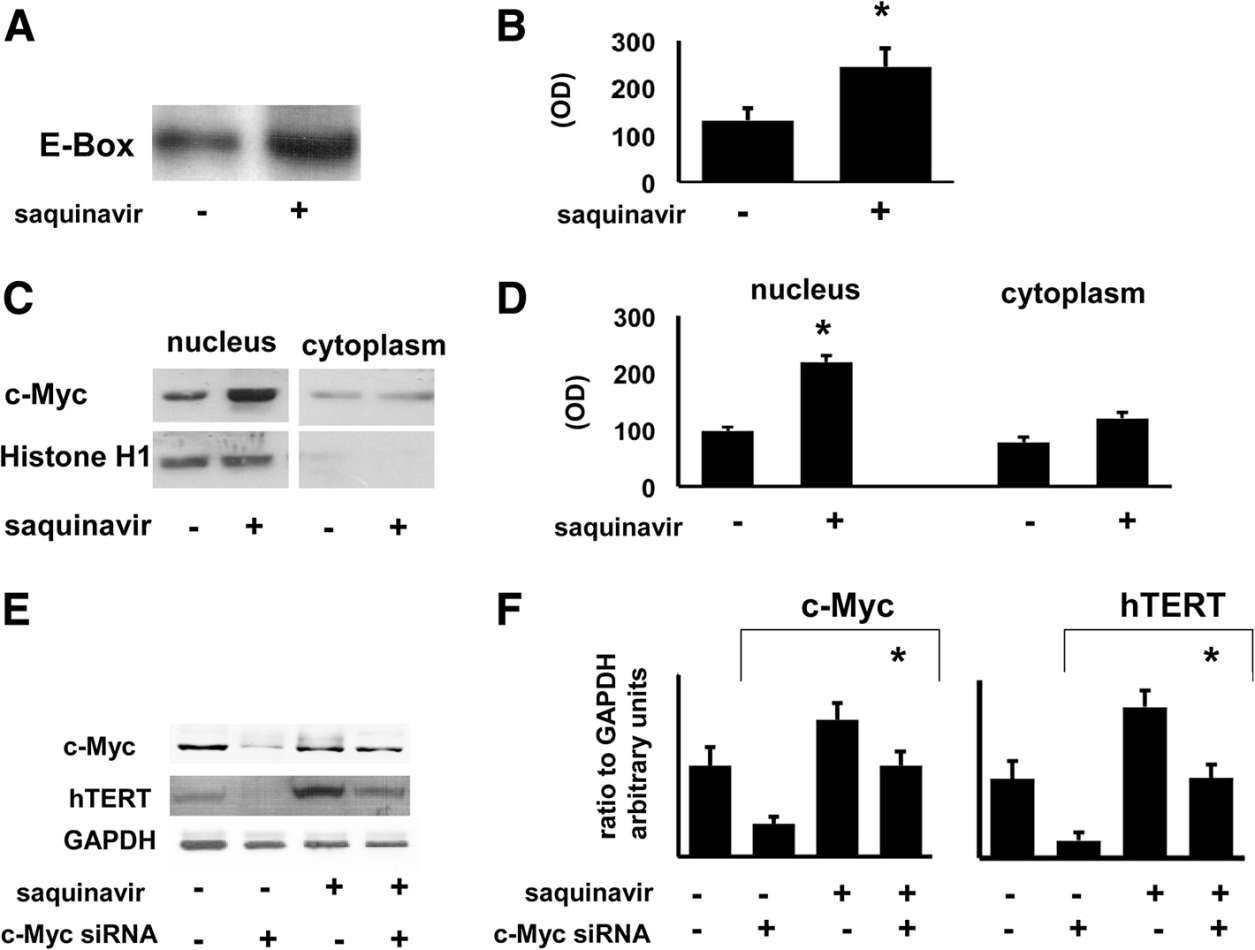
**Role of c-Myc in saquinavir activity. A**. Representative gel showing the binding of nuclear extracts of Jurkat cells to the oligonucleotide 5’- TCCTGCTGCGCACGTGGGAAGCCCT-3’, containing the downstream “CACGTG” E-Box sequence localized at position −34 of hTERT promoter, 24 h following exposure to saquinavir determined using EMSA. Saquinavir up-regulates the binding of nuclear proteins to the E-Box sequence. **B.** Graph shows the mean ± SD of the OD obtained from 3 EMSA independent experiments. **C.** Representative experiment showing the effect of saquinavir on c-Myc transcription factor expression tested on nuclear and cytoplasmic extracts of 2×10^6^ viable Jurkat cells after 24 h of treatment (Western Blot). Quality of nuclear extracts was tested using anti Histone H1 Ab. **D.** Graphs show the mean ± SD of c-Myc OD values obtained from 3 experiments of and all p values were calculated using Student’s t-test. **E.** Representative experiment showing the role of c-Myc on saquinavir-mediated hTERT up-regulation. Jurkat cells were transfected with siRNA targeting c-Myc mRNA as described in Material and Methods. c-Myc silencing induces marked down-regulation of c-Myc protein and hTERT which is a target of the transcriptional factor. Saquinavir restores c-Myc and hTERT expression to control levels. **F**. Pooled results relative to 2 separate experiments of c-Myc silencing. Asterisk indicates p < 0.05.

To further elucidate the involvement of a transcriptional mechanism underlying saquinavir- mediated increase of telomerase activity, we analyzed the binding of nuclear factors to the downstream E-Box sequence extrapolated from hTERT promoter, which is specific for c-Myc, the principal responsible for transcriptional activity of hTERT gene [21]. Saquinavir treatment was able to increase the binding of nuclear proteins to the E-Box sequence (Figure 3A). Pooled data from 3 different experiments confirmed the positive modulation of saquinavir on the binding to the E-Box portion of hTERT promoter (Figure 3B). To explore the role of c-Myc in saquinavir-mediated up-regulation of hTERT transcription, we analyzed the effect of the protease inhibitor on the expression and cellular distribution of the oncogene product, principal responsible for hTERT gene transcription. We found that saquinavir increased c-Myc expression in the nuclei of saquinavir-treated Jurkat cells (Figure 3C). This observation strongly supports a role for this transcription factor in saquinavir mediated up-regulation of hTERT levels and telomerase activity in Jurkat cells. Results relative to 3 separate experiments are shown in Figure 3D.

### Role of c-Myc in saquinavir-induced hTERT up-regulation

In order to better understand the role of c-Myc in the observed saquinavir-induced hTERT up-regulation, we transfected transiently Jurkat cells with siRNA targeting c-Myc mRNA. The results shown in Figure 3E point out that a marked down-regulation of c-Myc protein occurred in c-Myc silenced cells. On the other hand, combined treatment of target cells with saquinavir and siRNA restored c-Myc expression to control levels (Figure 3E). Interestingly, siRNA-mediated inhibition of c-Myc was followed by a marked decline of hTERT expression, which was restored by concomitant exposure to saquinavir (Figure 3E). Pooled results relative to 2 separate siRNA experiments are shown in Figure 3F.

## Discussion

The present report shows for the first time that an antiretroviral molecule belonging to PIs such as *s*aquinavir, is able to induce a rapid increase of telomerase activity in malignant cells of haematopoietic origin, while inhibiting their proliferative potential. In a number of different biological systems, telomerase activation is linked to increased cell proliferation and malignant cell aggressiveness [24]. However, in the case of saquinavir, our results did not show increased target cell proliferation, but rather cell inhibition. This in accordance with previous findings of other laboratories that demonstrated antitumor effects of this drug in different experimental models [3,4,12,25]. The inhibition of tumor cell growth and the pro-apoptotic effects of saquinavir have been linked to its suppressive activity on proteosoma [26], metalloproteases and neoangiogenesis [4]. All these effects appear to be mainly the consequence of saquinavir-induced impairment of Akt activation based on molecule phosphorylation [27].

In previous studies, we have shown that saquinavir is able to increase telomerase activity of normal peripheral blood mononuclear cells [8,9]. The present study extends this observation to Jurkat cells, a T leukaemia cell line. In the case of MNC, the results indicated that saquinavir increased telomerase activity either non-stimulated, or stimulated with PHA or with anti-CD3 plus anti-CD28 monoclonal antibodies. In our leukaemia model we revealed that drug-induced telomerase up-regulation was essentially due to increased expression and activation of the reverse transcriptase component (i.e. hTERT) of the enzyme complex. This has been found in terms of either increased hTERT mRNA and protein level. The mechanism underlying this effect appears to be related to the activation of hTERT gene promoter revealed by the increased binding of nuclear extracts of Jurkat cells to the E-Box sequence of the promoter, 24 h after exposure to saquinavir, as shown by EMSA analysis illustrated in Figure 3A. Previous studies performed by Furuya et al. [28], showed that survivin up-regulates hTERT expression through a cascade of intracellular signals starting from activation of Aurora B kinase that phosphorylates c-Myc which, in turn, in association with phosphorylated SP1, binds and activates hTERT promoter. In our hands, saquinavir was found to increase the expression of c-Myc, especially in the nuclear fraction of drug-treated Jurkat cells, thus suggesting that this could be at least one of the biochemical events responsible of telomerase activation. No data are presently available to ascertain whether saquinavir is involved in survivin circuit with activating function. However, this hypothesis does not find support from other investigations that demonstrated that two other PIs such as ritonavir [29] and nelfinavir [30] suppress rather than activate survivin expression. More attractive is presently the hypothesis that, saquinavir-mediated up-regulation of c-Myc expression, could be the consequence of drug-induced proteosoma impairment [26], resulting in the failure of c-Myc protein degradation [31]. Indeed, the drug is able to reverse also the decline of c-Myc protein following siRNA- mediated “knock down”. In line with this hypothesis, beside to a c-Myc mediated increase of hTERT transcription, we cannot rule out also that reduction of protein degradation could be partially involved in saquinavir-induced hTERT up-regulation.

Of particular interest is the finding that saquinavir-induced telomerase increase was followed by increased proliferation rate in activated normal mononuclear cells [9]. On the contrary, as shown in the present study, cell growth impairment occurred when Jurkat leukemia cells were subjected to similar experimental conditions. No data are presently available to identify the mechanism underlying the different responses to saquinavir between normal and malignant lymphoid cells. It is reasonable to assume that telomerase activity and cell proliferation can be disjointed processes differentially regulated in different types of cells. For example, dichotomy between telomerase activity and proliferation was demonstrated in highly differentiated “old” CD8^+^T cells following PDL-1 signalling blockade [32]. In any case, the finding that saquinavir is able to augment telomerase activity could be considered a negative aspect of the pharmacological profile of this molecule in oncology. However, high levels of telomerase are constitutively expressed in the majority of malignant cells (reviewed in 13). Therefore, increase of telomerase expression should not modify substantially the already “immortal” phenotype produced by the basal levels of this enzyme complex in cancer cells [33]. On the other hand, large experimental evidence is now available showing that hTERT could be involved in host’s immune responsiveness against autochtonous tumor. A number of HLA-restricted peptides can be generated following proteosomal-mediated degradation of hTERT protein. These peptides, presented by Class I HLA molecules on malignant cell surface elicit CD8^+^ T cell cytotoxic response of the host, leading to potentially efficient antitumor immunity (reviewed in 15, 16). It is reasonable to hypothesize that drug-induced up-regulation of hTERT could increase the probability of endocellular generation of hTERT-derived peptides showing the molecular pattern required for presentation in association with class I *HLA* gene products on the cell membrane of neoplastic cells. This would enhance, at least in principle, the level of host’s immune cytotoxic responsiveness against malignant cells. In fact, a similar mechanism has been found to take place with other tumor-associated antigens, such as CEA [34,35] or Thymidilate synthase [36] under the effect of 5-fluorouracil. However, observations showing that saquinavir could enhance c-Myc and possibly hTERT protein expression at least in part through proteasome activity inhibition seem to contrast the hypothesis that this agent could increase target cell immunogenicity. In fact, the presence of large amounts of immunogenic peptides requires substantial protein degradation via ubiquitin-proteasome system. Nevertheless, it is reasonable to assume that drug-induced protein accumulation could be followed by a “rebound” phenomenon, with augmented hTERT degradation and increased levels of hTERT-derived immunogenic peptides in target cells upon saquinavir withdrawal. Indeed, this type of antigen presentation kinetics is currently under investigation in our laboratory.

The observation that saquinavir increases c-Myc levels is in line with the finding that the drug is able to induce apoptosis [7,11]. Actually, c-Myc possesses a crucial function in controlling cell growth, differentiation and apoptosis, while its abnormal expression is associated with many tumors. Overexpression of c-Myc has been shown to sensitize tumor cells to apoptosis by amplifying the intrinsic mitochondrial pathway and by triggering the death receptor pathways by a variety of stimuli [37]. Therefore an hypothesis is that the intracellular accumulation of possibly polyubiquinated c-Myc following the saquinavir-mediated inhibition of the proteasome, could contribute to explain the mechanism underlying the apoptosis observed in different tumor cell models treated with the protease inhinibitor [7,11] and is currently under investigation.

## Conclusions

In conclusion, the present report shows for the first time that saquinavir is able to increase telomerase activity in leukaemia T cells, thus extending a similar finding previously obtained by us in normal haemopoietic cells to the area of haemato-oncology. Moreover, this study indicates c-Myc as molecular target of saquinavir, suggesting new perspectives in the pharmacological applications of PIs. On the other hand, in accordance with previous reports showing antitumor activity of saquinavir, we confirmed that the drug does not enhance but rather inhibited the growth of leukaemic cells. Therefore, saquinavir appears to play an attracting role as a potential antitumor agent, since along with its inhibiting effect on cell proliferation it could provide a novel strategy for increasing malignant cell immunogenicity.

## Competing interests

The authors declare that they have no competing interests.

## Authors’ contributions

All authors participated in the design, interpretation of the data and review of the manuscript. RA, AC, AA, LB, LG and OF performed the experiments. OF and EB wrote the manuscript. All authors read and approved the final manuscript.

## References

[B1] Andreoni M Perno CF Positioning of HIV-protease inhibitors in clinical practice Eur Rev Med Pharmacol Sci 2012 16 10 18 22338543

[B2] D’Alessandro A Pieroni L Ronci M D’Aguanno S Federici G Proteasome inhibitors therapeutic strategies for cancer Recent Pat Anticancer Drug Discov 2009 4 73 82 10.2174/157489209787002452 19149689

[B3] Monini P Sgadari C Toschi E Barillari G Ensoli B Antitumour effects of antiretroviral therapy Nat Rev Cancer 2004 4 861 875 10.1038/nrc1479 15516959

[B4] Toschi E Sgadari C Malavasi L Bacigalupo I Chiozzini C Human immunodeficiency virus protease inhibitors reduce the growth of human tumors via a proteasome-independent block of angiogenesis and matrix metalloproteinase’s Int J Cancer 2011 128 82 93 10.1002/ijc.25550 20617515

[B5] Donia M Maksimovic-Ivanic D Mijatovic S Mojic M Miljkovic D Timotijevic G In vitro and in vivo anticancer action of Saquinavir-NO, a novel nitric oxide-derivative of the protease inhibitor saquinavir, on hormone resistant prostate cancer cells Cell Cycle 2011 10 492 499 10.4161/cc.10.3.14727 21270522

[B6] Rothweiler F Michaelis M Brauer P Otte J Weber K Fehse B Anticancer effects of the nitric oxide-modified saquinavir derivative saquinavir-NO against multidrug-resistant cancer cells Neoplasia 2010 12 1023 1030 21170266 10.1593/neo.10856 PMC3003137

[B7] McLean K VanDeVen NA Sorenson DR Daudi S Liu J The HIV protease inhibitor saquinavir induces endoplasmic reticulum stress, autophagy, and apoptosis in ovarian cancer cells Gynecol Oncol 2009 112 23 630 10.1016/j.ygyno.2008.11.028 19147209

[B8] Franzese O Comandini FA Lombardi A Saponiero A Bonmassar E Saquinavir up-regulates telomerase activity in lymphocytes activated with monoclonal antibodies against CD3/CD28 J Chemother 2001 4 384 388 10.1179/joc.2001.13.4.384 11589480

[B9] Franzese O Lombardi A Comandini A Cannavò E Testorelli C Cirello I Effect of Saquinavir on proliferation and telomerase activity of human peripheral blood mononuclear cells Life Sci 2001 9 1509 1520 11554612 10.1016/s0024-3205(01)01243-7

[B10] Sgadari C Barillari G Toschi E Carlei D Bacigalupo I Baccarini S HIV protease inhibitors are potent anti-angiogenic molecules and promote regression of Kaposi sarcoma Nat Med 2002 8 225 232 10.1038/nm0302-225 11875492

[B11] Pajonk F Himmelsbach J Riess K Sommer A McBride WH The human immunodeficiency virus (HIV)-1 protease inhibitor saquinavir inhibits proteasome function and causes apoptosis and radiosensitization in non-HIV-associated human cancer cells Cancer Res 2002 62 5230 5235 12234989

[B12] Timeus F Crescenzio N Ricotti E Doria A Bertin D The effects of saquinavir on imatinib-resistant chronic myelogenous leukemia cell lines Haematologica 2006 91 711 712 16670078

[B13] Shay JW Wright WE Role of telomeres and telomerase in cancer Semin Cancer Biol 2011 21 349 353 10.1016/j.semcancer.2011.10.001 22015685 PMC3370415

[B14] Vonderheide RH Telomerase as a universal tumor-associated antigen for cancer immunotherapy Oncogene 2002 21 674 679 10.1038/sj.onc.1205074 11850795

[B15] Wenandy L Sorensen RB Sengelov L Svane IM Thor Straten P Andersen MH The immunogenicity of the hTERT540-548 peptide in cancer Clin Cancer Res 2008 14 4 7 10.1158/1078-0432.CCR-07-4590 18172245

[B16] Tian X Chen B Liu X Telomere and telomerase as targets for cancer therapy Appl Biochem Biotechnol 2010 160 1460 1472 10.1007/s12010-009-8633-9 19412578

[B17] Niu BL Du HM Shen HP Lian ZR Li JZ Lai X Myeloid dendritic cells loaded with dendritic tandem multiple antigenic telomerase reverse transcriptase (hTERT) epitope peptides: a potentially promising tumor vaccine Vaccine 2012 30 3395 3404 10.1016/j.vaccine.2012.03.045 22480929

[B18] Pepponi R Marra G Fuggetta MP Falcinelli S Pagani E Bonmassar E The effect of O6-alkylguanine-DNA alkyltransferase and mismatch repair activities on the sensitivity of human melanoma cells to temozolomide, 1,3-bis(2-chloroethyl)1-nitrosourea, and cisplatin J Pharmacol Exp Ther 2003 304 661 668 10.1124/jpet.102.043950 12538819

[B19] Wright WE Shay JW Piatyszek MA Modifications of a telomeric repeat amplification protocol (TRAP) result in increased reliability, linearity and sensitivity Nucleic Acids Res 1995 23 3794 3795 10.1093/nar/23.18.3794 7479015 PMC307284

[B20] Wang Z Kyo S Maida Y Takakura M Tanaka M Yatabe N Tamoxifen regulates human telomerase reverse transcriptase (hTERT) gene expression differently in breast and endometrial cancer cells Oncogene 2002 21 3517 3524 10.1038/sj.onc.1205463 12032853

[B21] Yagoa M Ohkia R Hatakeyamaa S Fujitab T Ishikawa F Variant forms of upstream stimulatory factors (USFs) control the promoter activity of hTERT, the human gene encoding the catalytic subunit of telomerase FEBS Lett 2002 520 40 46 10.1016/S0014-5793(02)02757-6 12044867

[B22] Andrews NC Faller DV A rapid micropreparation technique for extraction of DNA binding proteins from limiting numbers of mammalian cells Nucleic Acids Res 1991 19 2499 10.1093/nar/19.9.2499 2041787 PMC329467

[B23] Horikawa I Barrett JC Transcriptional regulation of the telomerase hTERT gene as a target for cellular and viral oncogenic mechanisms Carcinogenesis 2003 24 1167 1176 10.1093/carcin/bgg085 12807729

[B24] Hoos A Hepp HH Kaul S Ahlert T Bastert G Wallwiener D Telomerase activity correlates with tumor aggressiveness and reflects therapy effect in breast cancer Int J Cancer 1998 79 8 12 10.1002/(SICI)1097-0215(19980220)79:1<8::AID-IJC2>3.0.CO;2-5 9495350

[B25] Timeus F Crescenzio N Doria A Foglia L Pagliano S Ricotti E In vitro anti-neuroblastoma activity of saquinavir and its association with imatinib Oncol Rep 2012 27 734 740 22159894 10.3892/or.2011.1582

[B26] Piccinini M Rinaldo MT Anselmino A Buccinnà B Ramondetti C Dematteis A The HIV protease inhibitors Nelfinavir and Saquinavir, but not a variety of HIV reverse transcriptase inhibitors, affect adversely human proteosome function Antivir Ther 2005 10 215 223 15865215

[B27] Gupta AK Cerniglia GJ Mick R McKenna WG Muschel RJ HIV protease inhibitors block Akt signaling and radiosensitize tumor cells both in vitro and in vivo Cancer Res 2005 65 8256 8265 10.1158/0008-5472.CAN-05-1220 16166302

[B28] Furuya M Tsuji N Kobayashi D Watanabe AN Interaction between survivin and aurora-B kinase plays an important role in survivin-mediated up-regulation of human telomerase reverse transcriptase expression Int J Oncol 2009 34 1061 1068 19287963 10.3892/ijo_00000232

[B29] Srirangam A Milani M Mitra R Guo Z Rodriguez M Kathuria H The human immunodeficiency virus protease inhibitor ritonavir inhibits lung cancer cells, in part, by inhibition of survivin J Thorac Oncol 2011 6 661 670 10.1097/JTO.0b013e31820c9e3c 21270666 PMC3104055

[B30] Gupta V Samuleson CG Su S Chen TC Nelfinavir potentiation of imatinib cytotoxicity in meningioma cells via survivin inhibition Neurosurg Focus 2007 23 4 E9 10.3171/FOC-07/10/E9 17961046

[B31] Gregory MA Hann SR c-Myc proteolysis by the ubiquitin-proteasome pathway: stabilization of c-Myc in Burkitt’s lymphoma cells Mol Cell Biol 2000 20 2423 2435 10.1128/MCB.20.7.2423-2435.2000 10713166 PMC85426

[B32] Henson SM Macaulay R Franzese O Akbar AN Reversal of functional defects in highly differentiated young and old CD8^+^ T cells by PDL blockade Immunology 2012 135 355 363 10.1111/j.1365-2567.2011.03550.x 22211948 PMC3372750

[B33] Simsek BC Pehlivan S Karaoglu A Human telomerase reverse transcriptase expression in colorectal tumors: correlations with immunohistochemical expression and clinicopathologic features Ann Diagn Pathol 2010 14 413 417 10.1016/j.anndiagpath.2010.06.007 21074689

[B34] Prete SP Aquino A Masci G Orlando L Giuliani A De Santis S Drug-induced changes of carcinoembryonic antigen expression in human cancer cells: effect of 5-fluorouracil J Pharmacol Exp Ther 1996 279 1574 1581 8968385

[B35] Correale P Aquino A Giuliani A Pellegrini M Micheli L Cusi MG Treatment of colon and breast carcinoma cells with 5-fluorouracil enhances expression of carcinoembryonic antigen and susceptibility to HLA-A(*)02.01 restricted, CEA-peptide-specific cytotoxic T cells in vitro Int J Cancer 2003 104 437 445 10.1002/ijc.10969 12584740

[B36] Correale P Del Vecchio MT Di Genova G Savellini GG La Placa M Terrosi C 5-fluorouracil-based chemotherapy enhances the antitumor activity of a thymidylate synthase-directed polyepitopic peptide vaccine J Natl Cancer Inst 2005 97 1437 1445 10.1093/jnci/dji188 16204693

[B37] Hoffman B Liebermann DA Apoptotic signaling by c-MYC Oncogene 2008 27 6462 6472 10.1038/onc.2008.312 18955973

